# Telerehabilitation’s Safety, Feasibility, and Exercise Uptake in Cancer Survivors: Process Evaluation

**DOI:** 10.2196/33130

**Published:** 2021-12-21

**Authors:** Amy Dennett, Katherine E Harding, Jacoba Reimert, Rebecca Morris, Phillip Parente, Nicholas F Taylor

**Affiliations:** 1 Allied Health Clinical Research Office Eastern Health Box Hill Australia; 2 School of Allied Health, Human Services and Sport La Trobe University Bundoora Australia; 3 Department of Cancer Services Eastern Health Box Hill Australia; 4 Eastern Health Clinical School Monash University Box Hill Australia

**Keywords:** telehealth, exercise, telerehabilitation, physical activity, supportive care, COVID-19, feasibility, cancer, cancer survivor, evaluation, rehabilitation, impact, development, implementation

## Abstract

**Background:**

Access to exercise for cancer survivors is poor despite global recognition of its benefits. Telerehabilitation may overcome barriers to exercise for cancer survivors but is not routinely offered.

**Objective:**

Following the rapid implementation of an exercise-based telerehabilitation program in response to COVID-19, a process evaluation was conducted to understand the impact on patients, staff, and the health service with the aim of informing future program development.

**Methods:**

A mixed methods evaluation was completed for a telerehabilitation program for cancer survivors admitted between March and December 2020. Interviews were conducted with patients and staff involved in implementation. Routinely collected hospital data (adverse events, referrals, admissions, wait time, attendance, physical activity, and quality of life) were also assessed. Patients received an 8-week telerehabilitation intervention including one-on-one health coaching via telehealth, online group exercise and education, information portal, and home exercise prescription. Quantitative data were reported descriptively, and qualitative interview data were coded and mapped to the Proctor model for implementation research.

**Results:**

The telerehabilitation program received 175 new referrals over 8 months. Of those eligible, 123 of 150 (82%) commenced the study. There were no major adverse events. Adherence to health coaching was high (674/843, 80% of scheduled sessions), but participation in online group exercise classes was low (n=36, 29%). Patients improved their self-reported physical activity levels by a median of 110 minutes per week (IQR 90-401) by program completion. Patients were satisfied with telerehabilitation, but clinicians reported a mixed experience of pride in rapid care delivery contrasting with loss of personal connections. The average health service cost per patient was Aus $1104 (US $790).

**Conclusions:**

Telerehabilitation is safe, feasible, and improved outcomes for cancer survivors. Learnings from this study may inform the ongoing implementation of cancer telerehabilitation.

## Introduction

International guidelines promote exercise and rehabilitation as part of high-quality cancer care [1]. Exercise mitigates negative side effects of cancer treatment such as fatigue, improves physical function and quality of life, and is associated with reduced cancer recurrence and cancer-related mortality [2-4]. Despite compelling evidence to support exercise, it is not routinely integrated into standard cancer care.

Few specialized exercise-based rehabilitation programs exist for cancer survivors [5]. Cancer survivors experience unique issues related to their cancer management, which create barriers to exercise. These include treatment side effects such as fatigue; competing medical demands; and difficulties with travel, cost, and parking [6-8]. Telehealth may overcome these barriers by enabling patients to avoid additional travel, thereby conserving energy. In turn, this may increase their ability to access exercise support [9,10]. Cancer survivors describe telehealth as convenient, reassuring, and minimizing treatment burden [11]. Telehealth uses technologies such as videoconferencing, telephone, and mobile apps for diagnosis, treatment, and prevention of disease [12]. Telerehabilitation, a subfield of telehealth, improves patient outcomes in a variety of chronic diseases [13-17] and has been associated with improved mobility, fitness, and exercise adherence in cancer settings [18,19]. Reduced pain and shorter hospital length of stay with readmissions has also been reported for people with advanced cancer participating in telerehabilitation compared with usual care [17]. However, implementation of telerehabilitation remains limited in clinical practice.

A rapid uptake of telehealth to provide exercise for cancer survivors occurred during the COVID-19 pandemic due to social distancing restrictions [20]. There is sufficient evidence that telerehabilitation can work, but less is known about how it works in clinical settings. In contrast to trials, which aim to evaluate effectiveness, process evaluations provide information about how outcomes are reached, including barriers and facilitators to achieving an outcome [21]. Understanding implementation of telerehabilitation during the COVID-19 pandemic will help inform its broader implementation. Therefore, the aim of this study is to complete a process evaluation of an exercise-based cancer telerehabilitation program.

## Methods

### Study Design

A process evaluation using a mixed methods approach was completed to understand the implementation of cancer telerehabilitation within a subacute hospital setting. The Proctor model for implementation research provided an evaluation framework comprising a taxonomy of three categories (service, implementation, client) of which this study focused on eight key outcomes: safety, acceptability, adoption, feasibility, fidelity, cost, satisfaction, and quality of life [22]. This study used prospective and retrospective qualitative and quantitative data, and was reported according to the Strengthening the Reporting of Observational Studies in Epidemiology (STROBE) statement [23] and Template for Intervention Description and Replication (TIDieR) [24] checklist. Ethical approval was obtained from the hospital Human Research and Ethics Committee before participant recruitment commenced (LR20-045).

### Setting

The study was set in a large publicly funded health network in metropolitan Melbourne, Australia. The health network services approximately 3000 cancer survivors annually. A multidisciplinary, in-person group, exercise-based cancer rehabilitation program delivered in an ambulatory setting was replaced by a comprehensive telerehabilitation program in March 2020 due to COVID-19 restrictions. Prior to COVID-19, telehealth was not offered to patients. Pre–COVID-19, the cancer rehabilitation program included twice-weekly, 1-hour supervised group exercise and once-weekly multidisciplinary group education over 7 weeks. The average cost of the in-person program to the health service was Aus $1402 (US $1004) per patient (Aus $108 [US $77] per session), to which patients contributed Aus $140 (US $100).

### Intervention

The telerehabilitation program was an 8-week supervised program with multiple components delivered by a nurse coordinator, 3 physiotherapists, and an allied health assistant (Table 1). Patients completed a 1-hour comprehensive assessment via phone or videoconference (HealthDirect, Melbourne, Australia) and were offered weekly individual health coaching sessions and a scheduled, weekly, live, supervised, online group exercise and education class (Cisco WebEx, Milpitas, California; held sequentially on the same day). Patients also received access to an online portal (iLearn, Totara Learning Solutions, Wellington, New Zealand) and a home exercise program (Physitrack, London, United Kingdom). All patients were enrolled in scheduled health coaching sessions and were offered and encouraged to participate in all elements of the program but could choose whether to access the online group classes and information portal. Referrals to other professionals (occupational therapist, social worker, nurse, dietitian) were made as required. Clinical staff were trained by participating in three 3-hour online health coaching workshops (focused on motivational interviewing) and one 1-hour online information session on how to use the health network’s telehealth platform.

**Table 1:** Intervention description using the Template for Description and Replication Checklist (TIDieR) compared with the traditional program model.

		Telerehabiliation intervention	Traditional face-to-face model
Brief name		Cancer telerehabilitation	Cancer rehabilitation
Why		Telehealth replaced the traditional face-to-face model of care during COVID-19 restrictions for safety	Face-to-face exercise is the traditional modality of delivering cancer rehabilitation
What: materials		Health coaching (videoconference or telephone) Optional online group exercise (live videoconference via WebEx) Optional online group multidisciplinary education (live videoconference via WebEx) Written or app-based (Physitrack), individualized home exercise program and exercise band Online information portal (iLearn) with recordings of multidisciplinary education, information handouts, and weblinks or written information handouts Participants were offered a referral to a community exercise program on completion	Face-to-face group exercise with tailored exercise advice within group Optional face-to-face group multidisciplinary education Written individualized home exercise program Participants were offered a referral to a community exercise program on completion
**What procedures**			
	Provider	Two midlevel physiotherapists and one senior physiotherapist^a^ with oncology experience employed by the hospital One senior oncology nurse employed by the hospital One allied health assistant provided by the hospital One administration assistant	Two midlevel physiotherapists with oncology experience employed by the hospital One senior oncology nurse employed by the hospital One allied health assistant provided by the hospital One administration assistant
	How	Supervised sessions via telephone or videoconference	Face-to-face supervision
	Where	Clinicians: hospital based; patients: home based	Clinicians and patients: hospital based
**When/how much**			
	Type	Aerobic: walking, aerobics, step-ups Resistance: exercise bands, body weight exercise, free weights^b^ Flexibility: included as required based on individual needs	Aerobic: treadmill walking, stationary cycle, step-ups Resistance: exercise bands body weight exercise, free weights, cable weights machine Flexibility: included as required based on individual needs
	Intensity	Aerobic: moderate (BORG 3-4) Resistance: 2-3 sets 10-12 repetitions	Aerobic: moderate (BORG 3-4) Resistance: 2-3 sets 10-12 repetitions
	Frequency	1x weekly health coaching 1x weekly online group supervised training 1x weekly group education	2x weekly face-to-face group exercise 1x weekly face-to-face group education
	Session time	30-minute 1:1 health coaching reviews 45-minute online exercise group (live) 45-minute online education group (live)	60-minute face-to-face group exercise 45- to 60-minute face-to-face group education
Overall duration		8 weeks^c^	7 weeks
Tailoring		Individualized exercise program based on initial consultation and goals	Individualized exercise program based on initial consultation and goals
Trial fidelity		Staff with a background in oncology physiotherapy and nursing who had prior formal training were employed by the hospital to provide the intervention Motivational interviewing training (9 hours) and telehealth information session (1 hour) for clinical staff Electronic exercise log via Physitrack app Electronic records of the number and duration of completed sessions Clinical supervision as per standard hospital policy	Staff with a background in oncology physiotherapy and nursing who had prior formal training were employed by the hospital to provide the intervention Paper-based exercise logs to record number and duration of completed sessions Clinical supervision as per standard hospital policy

^a^Senior physiotherapist completed some similar duties to senior nurse (eg, patient intake) as hours of the nurse were reduced during the COVID-19 period.

^b^Exercise type may have differed depending on patient’s own equipment availability.

^c^Duration of program increased to better align with current evidence and other cancer rehabilitation programs.

### Participants

Patients were referred and admitted to the telerehabilitation program between March 23 and December 1, 2020. Patients who were referred to the oncology rehabilitation program prior to March 23 and transitioned from an in-person program, and who received more than one telerehabilitation session were also included in the analysis. To be eligible, patients had to be adult cancer survivors currently receiving or within 12 months of cancer treatment (curative or palliative intent). Patients with a cognitive impairment or receiving end of life care were excluded from the program. Patients may have been referred to an alternative rehabilitation service offering more supervision if they had recently been discharged from the hospital or had higher functional needs (eg, Australian Karnofsky Performance Status <60) in line with existing service criteria. For routinely collected data, individual patient consent was not sought, as the clinical members of the research team would normally have access to these data. Consent for postprogram data was implied through completion of an online survey, which included a participant information sheet.

Clinicians, administration staff, and managers directly involved in the implementation of the cancer telerehabilitation program were invited to participate in an interview. Staff participating in interviews provided written informed consent.

### Outcome Measures

Data were collected from a variety of sources (Multimedia Appendix 1).

#### Interviews

Staff were invited to participate in either a 1-hour focus group or 1:1 interview at the conclusion of program implementation to discuss their perceptions and experience of delivering the telerehabilitation model. These discussions focused on areas of safety, acceptability, adoption, feasibility, fidelity, and costs (Multimedia Appendix 2).

#### Survey

Patient perceptions and experiences of telerehabilitation were collected via an online survey (QuestionPro, Dallas, Texas) or telephone following the conclusion of the program to determine acceptability, feasibility, and satisfaction. The survey included the System Usability Scale, a 10-item questionnaire measuring usability with five response options (strongly agree to strongly disagree) [25]. Four open-ended questions were included in the survey asking patients about the benefits and challenges of telerehabilitation, how it compares to in-person rehabilitation, and general comments.

#### Routine Service and Outcome Data

Safety was assessed by recording adverse events from the medical record. Other routinely collected service data, including participant demographics (including physical performance score [26]), were collected to describe the sample. Acceptability, feasibility, and fidelity were assessed by reviewing referral, admission, wait time, and attendance data.

Routine patient-reported outcome measures described the feasibility and client outcomes. These included health-related quality of life (EQ-5D [27]), total physical activity time (Active Australia Survey [28]), and sedentary behavior (International Physical Activity Questionnaire sitting items [29]), which were collected at program entry and completion by physiotherapists delivering the program.

An analysis of session content documented in the medical record further assessed safety, feasibility, and fidelity. Routinely collected online metadata from the iLearn platform also informed feasibility and fidelity.

#### Cost Data

Program costs of the traditional and telerehabilitation model were derived from the calculation of staff salaries in line with industrial agreements and estimates of software costs for delivery of telehealth obtained from the organization’s information technology department. An average cost per patient, per session was calculated using the total admitted patients and total program costs.

### Data Analysis

Patient characteristics, adherence, safety, costs, and satisfaction were reported descriptively. Completers were defined as patients who completed at least 50% of the health coaching sessions (4 sessions). Only completers with completed postprogram measures were included in the analysis of client outcomes. Pre- and postpatient outcome data are reported using means and SDs calculated from normally distributed data and medians and IQRs for nonnormally distributed data. Within-group changes were calculated using Wilcoxon signed rank tests, as data were not normally distributed.

The content of the open-ended survey comments were coded and grouped into themes by two researchers independently using an inductive approach. Interviews were audio recorded and transcribed verbatim. Transcripts were deidentified and assigned an identification number to ensure anonymity. Transcripts were read and independently coded line by line by three authors (AD, NFT, JR) and using open coding (ie, the codes emerged from the data). Codes were categorized and discussed until consensus was reached on themes that were then mapped deductively onto the Proctor model.

A content analysis of telerehabilitation sessions was completed from a random sample of medical records from 50 patients. The documentation recorded in the medical record was assessed against predetermined criteria (Multimedia Appendix 3) to determine whether telerehabilitation interventions were delivered using behavior change interventions consistent with the principles of health coaching [30]. Data were analyzed using SPSS version 26 (IBM Corp).

## Results

### Participant Characteristics

**Figure 1:**
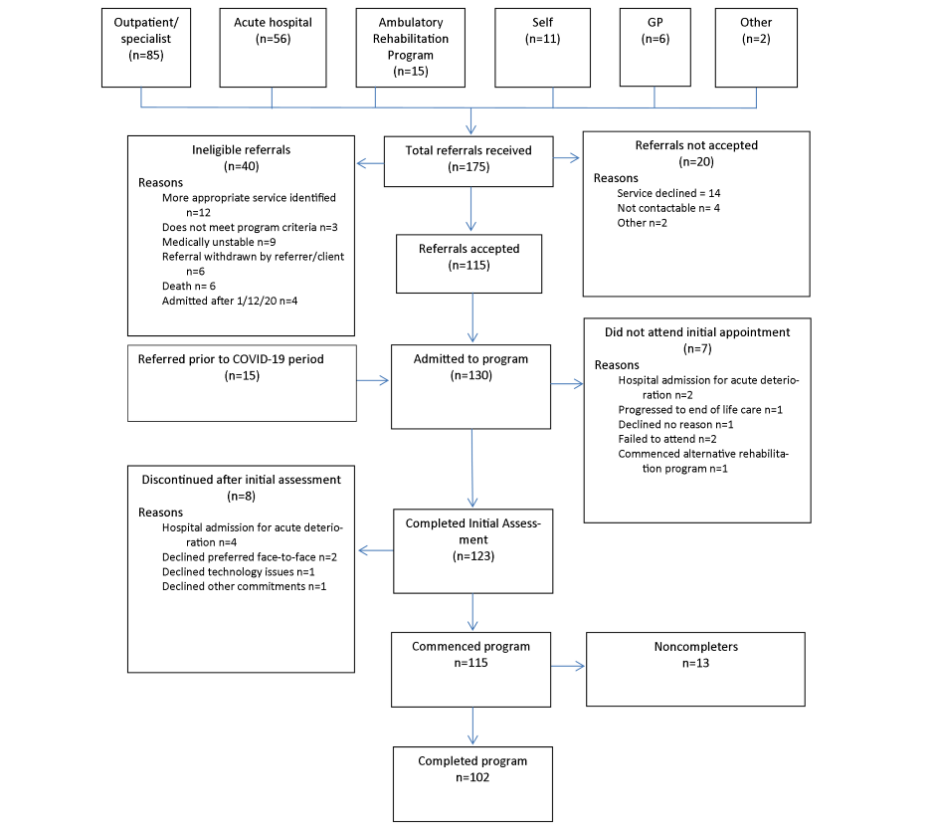
Flow of referrals. GP: general practitioners.

During the 8-month data collection period, 175 new referrals were received, most from oncology/hematology outpatient specialist clinics. Of the eligible referrals, 123 patients (including participants referred prior to COVID-19) commenced the program, representing 82% (123/150) uptake (Figure 1). The median wait time from referral to first appointment was 16 (IQR 9-28) days. The telerehabilitation modality of choice at admission was videoconference (93/123, 76%). A total of 102 (83%) participants completed the program.

Patients admitted to the program on average were aged 65 (IQR 56-72) years and 57% (n=66) were female. The most common diagnosis was breast cancer (n=39, 32%), followed by multiple myeloma (n=17, 14%). A total of 74 (60%) patients had advanced cancer, and 85% (n=104) were receiving treatment on admission to the program with the primary treatment being chemotherapy (n=69, 56%; Table 2). Patients had a median performance score of 70, indicating an inability to carry on usual work due to their disease. They lived a median of 12 (range 4-138) km from the hospital.

**Table 2:** Patient demographics.

Characteristic		Patient (N=123)
Age (years), median (IQR)		65 (56-72)
Gender (female), n (%)		66 (57)
Distance from hospital (km), median (range)		12 (4-138)
AKPS^a^ (0-100), median (IQR)		70 (70-80)
**Type of cancer, n (%)**		
	Breast	39 (32)
	Lower gastrointestinal	7 (6)
	Prostate	8 (7)
	Gynecological	6 (5)
	Multiple myeloma	17 (14)
	Lymphoma	10 (8)
	Leukemia	10 (8)
	Lung	7 (6)
	Other	19 (15)
**Cancer stage, n (%)^b^**		
	Early	40 (33)
	Advanced	74 (60)
	Recurrent	7 (6)
**Current treatment received, n (%)**		
	Chemotherapy	69 (56)
	Radiotherapy	18 (15)
	Immunotherapy	5 (4)
	Stem cell transplant	4 (3)
	Hormone therapy	6 (5)
	Other	2 (2)
	None	19 (15)

^a^AKPS: Australian Karnofsky Performance Status.

^b^Cancer stage not available for 2 participants.

### Service Outcomes

#### Safety

No major adverse events were attributed to the telerehabilitation program. Musculoskeletal pain or strain was the most reported minor adverse event (n=27). One patient had a noninjurious fall while completing their home exercise program unsupervised, another fell while walking (outside of the program) resulting in a hand fracture, but these events did not limit ongoing program participation. Five patients fell unrelated to exercise. One patient developed new lymphedema during the program.

A total of 12 patients experienced disease progression after program admission. Overall, 16 patients were admitted to the hospital during the program (3 due to falls unrelated to exercise, 6 due to disease progression, 7 due to other medical event), with 4 unable to continue beyond initial assessment and 3 unable to continue their rehabilitation program on discharge from hospital. Two patients died from an acute medical deterioration unrelated to program participation.

Overall, telerehabilitation was perceived as safe but staff acknowledged difficulty balancing safety needs with providing an adequate exercise prescription. Perception of safety was increased when patients used video. Staff also expressed reservations related to their competency to provide telehealth safely due to the rapid transition (Multimedia Appendix 4).

I think in terms of fitting with the model, the key difference [with telerehabilitation was] of safety and clinicians being able to monitor or assess their technique or how they're responding to the exercise.

Participant 3

### Implementation Outcomes

#### Acceptability

Surveys were returned by 82 cancer survivors (67% response rate). A total of 7 staff (3 physiotherapists, 1 nurse, 1 allied health assistant, 2 administration staff, mean experience 13 years) participated in a focus group, and 1 manager was interviewed.

The program was acceptable to both patients and clinicians. The median score on the System Usability Scale was 77.5 (IQR 67.5-90), indicating above average usability of telerehabilitation.

Staff described implementation of the program as a *rollercoaster.* The program was largely viewed by staff as a positive and acceptable form of delivering care. The team described pride in being able to deliver an innovative model of care in a short time frame:

They [the team] all see it as a positive...all of them are quite proud of what they've achieved...

Participant 8

The manager highlighted the value of the program’s flexibility, and all staff perceived it to be convenient for patients. However, the positives of telerehabilitation were counterbalanced by challenges of this new service delivery mode.

Staff felt isolated from each other and patients, and reflected on the importance of personal connections. Nonphysiotherapy staff felt a loss of connection with patients, while physiotherapists described a strengthening of patient connections. The whole team felt disconnected from each other, emotionally drained, and missed the dynamic group environment of the traditional rehabilitation model.

#### Adoption

Program staff described being impressed with the rapid transition to telehealth. Clinical and administrative staff attributed the success of the implementation to the combined efforts of the team, including their organizational, technical skills, and can-do attitude:

It was quite a rapid COVID force transitioning to this model...amazing how hurdles were jumped...There was very much a can-do mindset, from the team I think, across the board.

Participant 2

There was desire from all staff to continue with telerehabilitation into the future. The manager described the need for this model to be translated to other rehabilitation settings although questioned whether implementation of telerehabilitation in other chronic disease programs within the health service would be as successful as cancer rehabilitation who they perceived to comprise a cohort of younger patients in better health. This view contrasted with clinical staff who described a challenging cohort deconditioned with advanced cancer.

The main concern with ongoing adoption of the telerehabilitation model was from clinicians, who perceived that existing resources may be insufficient to provide the time and staff required to implement the model long term.

#### Feasibility

Staff acknowledged telehealth could be implemented in a cancer rehabilitation setting and described being seen by others within and beyond the organization as exemplars for telerehabilitation. They described the advantage of accessing existing supports that facilitated the transition. This included the organization’s existing telehealth platform and remote information technology support. However, clinicians at times felt underprepared to deliver telerehabilitation and wanted more guidance:

What we were giving our patients was safe and effective but then that was the tip of the iceberg...all the way below was all these other systems and processes that we had to get our heads around...

Participant 3

One of the main challenges of the program described by participants was poor internet infrastructure and lack of private space to complete online consults. Participants had difficulty accessing rooms for teleconferencing as they were shared with other programs within the hospital. There was also poor Wi-Fi coverage within certain hospital areas. Staff described the benefits of having hardware but that it was not helpful when the internet did not work.

#### Fidelity

All patients received health coaching from a physiotherapist. A total of 61 (50%) patients received at least one nursing session. A total of 17 patients were referred to other disciplines from supporting programs (4 participants received multiple referrals: 9 occupational therapy, 8 dietetics, 2 physiotherapy, 1 pharmacy, 1 social work). Most sessions were conducted via videoconference (n=381, 55%), followed by telephone (n=294, 42%), with the remaining sessions conducted in person. The average individual telehealth session duration was 25 (SD 9) minutes. Patients attended 80% (674/843) of scheduled 1:1 telehealth sessions. The primary reasons for nonattendance were unable to contact/forgot (90/169, 53% missed sessions), followed by conflicting appointments (37/169, 22% missed sessions). Of the 50 patients included in the retrospective file audit, 44 (88%) received a home exercise program. Behavior change interventions were used by physiotherapists in all 1:1 consults. Goal setting was the most used intervention (46/50, 92%), followed by demonstration (37/50, 74%) and evoking change talk (motivational interviewing; 28/50, 56%).

A total of 36 (29%) patients attended all telerehabilitation components at least once (group education, exercise, and 1:1 telerehabilitation). A total of 61 (50%) participants accessed the online portal at least once. The exercise webinar received the most views (n=40), followed by advanced care planning (n=34). A total of 18 (15%) patients attended >50% of online group exercise sessions. In the file audit, 19 of 50 (38%) patients attended online group exercise, and 17 of 50 (34%) attended live online group education. The most frequently attended live online education session was from the dietitian (10/50, 20%).

Physiotherapists perceived the program was effective for some patients, particularly those who engaged well with technology. However, they described a preference for delivering in-person care, as they felt more able to assess, monitor, and correct exercise prescription. Patients also described exercise monitoring as a key advantage of in-person care. Staff described adequate resourcing as essential to effective telerehabilitation delivery (Multimedia Appendix 4):

It’s hard knowing that they might not get, as much benefit...because ideally they would push a bit harder but just from a safety perspective. I didn’t want them to.

Participant 4

#### Costs

There was no cost to patients receiving telerehabilitation. Three patients required a home visit due to safety concerns, and 8 participants attended sessions at the center, as the program transitioned in and out of COVID-19 restrictions at a cost of Aus $10 (US $7) per in-person visit.

The primary resource cost was funding of staff (Multimedia Appendix 5). Existing telephone, internet connection, and software were used. Additional software for groups and equipment were purchased using a mix of internal and external funds. The average cost to the health service per patient for the program was Aus $1104 (US $790), equating to Aus $69 (US $49) per session per patient (assuming twice-weekly participation).

There were differing perceptions about the costs of telerehabilitation among staff. The manager described minimal costs associated with program setup and perceived efficiency in the new model. In contrast, clinicians described telerehabilitation as resource intensive compared to the previous group program due to perceived higher human resource costs from additional administrative burden of program setup and delivery, and the 1:1 nature of consults:

If they understood the funding requirement to get the throughput they want they couldn’t possibly support it.

Participant 4

### Client Outcomes

#### Satisfaction

Overall, 71 of 80 patients surveyed were satisfied with the telerehabilitation program, and 65 of 79 patients surveyed thought their health and well-being improved. Patients rated their confidence to continue exercising after the program positively (average 8/10). A positive experience was reported by most users in the open-ended responses (Textbox 1):

I am so impressed by the wonderful support the team gave me. It was unexpected but truly made a huge difference in my wellness journey.

Most benefits of the telerehabilitation program related to general support provided by the program. Patients commented frequently on their positive interactions with staff, who were described as helpful, friendly, and knowledgeable. Patients enjoyed learning new information to aid their recovery, especially related to exercise. The main challenge of the program was technology difficulties such as poor internet connection or audio-visual feedback. Other challenges were personal barriers related to their medical status including low motivation and fatigue.

**Benefits and challenges of telerehabilitation (selected patient quotes).**

**Benefits of telerehabilitation**

Convenience and efficiency

- “Telehealth means no driving or paying for parking which is good”
- “Better use of time, because I could do it when it suits me”
- “Telerehabilitation is my preferred option for its convenience. The easier it is, the more likely I am to participate”
- “I loved the whole experience. You could exercise at your own pace and I was able to conserve the energy usually taken up by getting in and out of the car for the appointment to be able to exercise more efficiently”

Safe

- “It was better because I did not get exposed to COVID, but I could still connect to people, which was very important during these times, especially in lockdown and I was isolated”
- “Obviously being immunocompromised after transplant not having to travel for physio and risk exposure was beneficial”
- “It is a very good alternative to in-person rehabilitation, when in-person is not feasible”

Communication

- “liked reading the followup notes - did a few days ahead of appointment prepare”
- “Pictures and clear instructions were easy to follow. The online tutorials were helpful as well”
- “I found [Physio] very easy to talk to and she was able to explain the various exercises clearly and concisely”

General clinician support and understanding

- “the support of the clinicians and the professionalism, everyone answered my questions”
- “being able to have someone to talk to as to how I was feeling and understood where I was at particularly during COVID lockdown”
- “probably the tailored exercises and having an excellent physiotherapist who listened and understood my issues”

Access to friendly, knowledgeable staff

- “being able to see the rehab specialist smiling face”
- “The weekly chats/inspiration with [physio] and her practical solutions”
- “Focussed presenters and knew their subject”

Gained motivation

- “It has been just excellent for me, the physiotherapist talked me through the program, it was had the biggest impact on me because I was lazy and not exercising due to my diagnosis and COVID. The program gave me the motivation to exercise and made me feel good and confident to exercise with cancer”
- “The weekly checkins and being able to talk to someone made me accountable. I felt inspired, [Physio] was super and had good tips”
- “Weekly contact made me supported and motivated”

Learning new things

- “Every week was outstanding, I learnt new things and all my questions were answered”
- “Having one person to discuss the benefits and advice given when needed, also having someone there for clarification. The education program also was very beneficial especially the Pharmacist session”
- “Learning how to exercise and I could feel myself getting stronger”

Access to exercise

- “It was good to make me exercise while I was incapacitated”
- “The demonstration of the exercises is useful and the introduction to other forms of exercise such as Tai Chi and Feldenkrais were great. It encouraged me to look these forms of exercise on Youtube”
- “Suggestions of exercises that I would not have thought of myself”

Personalized care

- “Targeted exercises for my special needs”
- “personal involvement in my rehab prior to my return to regular gym”
- “Individualised care and exercise program adjustments”

**Challenges of telerehabilitation**

Lack of social interaction

- “I enjoy the social interaction and seeing the person so I think I would have gotten a lot more out of attending a group at the centre”
- “The physical presence provides other support, like motivation, conversation, interaction (social), and spontaneous reaction with the physiotherapist and fellow participants, leading to a more relaxed environment”
- “I feel more motivated if I have to go to the centre, it gets lonely having to do exercise by yourself”

Issues with fidelity

- “It is different because your movements are not checked by a qualified person rather, it’s just shown to you via a video. So if a movement is incorrectly performed it’s not corrected”
- “1:1 and in person is better because someone can monitor you in real time”
- “Not attending on site and having access to the additional exercise equipment”

Technology difficulties

- “I am not proficient at using the computer so found it tricky to get into the program at times”
- “Phone reception terrible but otherwise ok”
- “Variable connectivity in telehealth sessions”

Lack of audio/visual feedback

- “some exercises were hard over the phone”
- “Not getting feedback about how I was performing and making adjustments, corrections, changes when appropriate. Sometimes it was difficult to see and hear the exercise performed- distance from the camera of the person performing the exercise/quality of the microphone...”
- “I am wary about services where the clinician cannot see the patient, you lose some input”

Managing symptoms

- “Became challenging to do rehab as pain increased”
- “I have been attending other health appointments through the phone as well and I get tired talking over the phone. I did not get to exercise in a group with other people”
- “Sometimes I had low energy to participate in the classes”

Low motivation

- “It had a big impact on my mental health, I was not motivated and I felt isolated”
- “Being inconsistent with the exercises during lock down. It's funny how excuses seem to infer with the exercise program and at times medical conditions interfere as well”
- “Daily exercising in house is hard. My day is busy with household activities”

#### Quality of Life

Health-related quality of life improved on the EQ-5D VAS (Z=–3.504; *P*<.001). There was no change in EQ-5D index scores (Z=–0.624; *P*=.53).

#### Physical Activity

From available data, 39% (n=43) of patients were meeting recommended physical activity levels at baseline, completing a median 100 (IQR 20-240) minutes of moderate-to-vigorous physical activity per week. By the end of the program, 65% (n=57) of patients met recommended physical activity levels, completing a median of 210 (IQR 90-401) minutes of moderate-to-vigorous physical activity per week (Z=–4.896; *P*<.001). Reported sedentary behavior decreased from 7.5 (IQR 5-10) hours per day to 6 (IQR 4-8) hours per day (Z=–2.301; *P*=.02).

## Discussion

### Principal Findings

This process evaluation demonstrated that a comprehensive telerehabilitation program is safe and feasible to improve health outcomes for cancer survivors. There was good program uptake and adherence to individual telehealth sessions, which was facilitated by convenience. Patients reported high satisfaction and ease interacting with telerehabilitation. Staff also described a positive experience with telerehabilitation, but this was counterbalanced by emotional fatigue and loss of personal connections. This process evaluation provides a practical outline of how telerehabilitation can be implemented and guidance for future development of cancer rehabilitation programs.

Telerehabilitation is an acceptable and feasible alternative to in-person care. Patients described the program as easy to use despite technical difficulties, and many would opt for a similar model in the future. Program satisfaction came from emotional and practical support rather than factors related to the modality of training. Key benefits related to interactions provided by staff, consistent with traditional models of cancer rehabilitation [31]. However, specific components of the telerehabilitation program appeared less feasible, which may affect overall effectiveness. For example, there was low uptake of the online portal and online group classes, which may lower the effectiveness of telerehabilitation if patients are not exercising independently outside of therapy time. No trials have been conducted evaluating online group exercise classes for cancer survivors [32], but it is well known that supervised in-person exercise improves cancer outcomes compared to usual care [2]. Hybrid models of cancer rehabilitation including telehealth could be considered to allow patients choice and improve access to exercise for cancer survivors.

Telerehabilitation may help facilitate access to exercise for cancer survivors. Uptake was higher, and adherence to 1:1 telehealth sessions was comparable to in-person cancer rehabilitation delivered in nonresearch settings [33-35]. Patients and clinicians highlighted convenience as a strength of telerehabilitation, consistent with previous literature [11]. Patient challenges with program participation related to personal factors such as motivation, fatigue, and other medical issues, similar to in-person rehabilitation [6,7,36]. Telerehabilitation offers an opportunity to participate in exercise by minimizing disruption and allowing cancer survivors to exercise at their own pace consistent with their desire to access convenient exercise rehabilitation programs, especially during treatment [6,37]. By increasing access and encouraging exercise adherence through telerehabilitation, there is also opportunity for lower health care expenditure in addition to improved patient outcomes [38].

Cost-effectiveness data for cancer telerehabilitation is lacking [9]. In this evaluation, costs of telerehabilitation were lower than the previous in-person rehabilitation model at this health service and similar to other published in-person models of cancer rehabilitation [39-41]. Costs may be lower for maintenance of telerehabilitation programs with additional cost savings for telerehabilitation programs realized over time, as setup costs are absorbed and the need for on-site premises reduces. During the implementation period, the service managed a higher rate of demand, more 1:1 consultations, and a lower staff to patient ratio for online groups with similar staffing levels. These observations are likely to explain why clinical staff perceived higher resource cost with telerehabilitation, emphasizing the need for strategies to support staff when changing practice such as engagement and feedback [42]. These perceptions were in the context of a reported loss of team connection, further highlighting the importance of nonclinical duties such as meetings and team-building activities. Given that costs are a key driver of decision-making in health care, more work is required to evaluate the cost-effectiveness of telerehabilitation to inform its wider implementation.

Patients made clinically significant improvements in self-reported physical activity levels. At baseline, patients completed a median of 100 minutes of moderate-to-vigorous physical activity per week, while at program completion, patients completed a median of 210 minutes per week, exceeding physical activity recommendations. This is noteworthy given that low physical activity is a problem in people receiving cancer rehabilitation [43] and that improving physical activity through group exercise rehabilitation alone is difficult [44]. Health coaching that intentionally included behavior change techniques in the telerehabilitation model in lieu of offering regular in-person group exercise may have contributed to this improvement. This finding was consistent with recent reviews of telehealth demonstrating improvements in physical activity levels of cancer survivors [45,46]. Telehealth may be a feasible way to supplement traditional exercise-based rehabilitation programs to encourage long-term participation in physical activity.

### Strengths and Limitations

To our knowledge, this is the first study to conduct a process evaluation with an exercise-based cancer telerehabilitation program. This study was reported in accordance with STROBE and TIDieR guidelines, which will assist replication of findings in other cancer settings. A strength of this research increasing generalizability is that it evaluates a pragmatic program in a public hospital setting, including older people and those with advanced cancer who are frequently omitted from exercise oncology research.

A limitation of this study is that it includes a relatively small nonrandomized sample of patients with a risk of selection bias. However, a broad demographic of cancer survivors was represented, and the inclusion of telephone interventions ensured access to the program would not be limited to patients with internet. A limited cost analysis was completed that did not consider patient, travel, or infrastructure costs, which may underestimate the value of telerehabilitation. Physical activity levels in this study were measured by self-report and therefore are subject to recall bias. In addition, no outcomes from the in-person program were available for comparison, as routinely collected outcome measures were changed in response to the change in program delivery. However, the primary aim of this study was not to demonstrate efficacy but rather to understand implementation to guide future models of cancer rehabilitation.

### Conclusions

This study demonstrated that exercise-based cancer rehabilitation delivered by telehealth is safe, feasible, and accepted by patients. Clinicians reported a mixed experience with telerehabilitation implementation, describing it as a *rollercoaster*. Our findings demonstrate telerehabilitation is affordable and can be translated pragmatically and quickly into hospital settings, which may improve access to exercise for cancer survivors. However, staff implementing telerehabilitation programs need adequate support. Further research is required to confirm the efficacy and cost-effectiveness of exercise-based telerehabilitation programs, so they can be integrated into standard care.

## Appendix

Multimedia Appendix 1 Data sources for each outcome measure.

Multimedia Appendix 2 Interview questions.

Multimedia Appendix 3 Criteria for fidelity audit.

Multimedia Appendix 4 Selected interview quotes.

Multimedia Appendix 5 Telerehabilitation program costs.

## Abbreviations

STROBE: Strengthening the Reporting of Observational Studies in Epidemiology
TIDieR: Template for Intervention Description and Replication
