# Supplementary material for: Telerehabilitation’s Safety, Feasibility, and Exercise Uptake in Cancer Survivors: Process Evaluation
Source: JMIR Cancer. 2021 Dec 21;7(4):e33130. doi: 10.2196/33130 (PMC8768007; doi:10.2196/33130)
Supplement: Multimedia Appendix 1 [file cancer_v7i4e33130_app1.docx]

Supplementary File 1. Data sources for each outcome measure

| **Outcome** | **Staff interviews** | **Survey** | **Manager Interviews** | **Recruitment Data** | **Routine service data** | **Routine PROMS** | **Analysis of sessions** | **Cost data** | **iLearn Data** |
| --- | --- | --- | --- | --- | --- | --- | --- | --- | --- |
| **Service Outcome**  Safety | ✓ |  | ✓ |  | ✓ |  | ✓ |  |  |
| **Implementation Outcomes**  Acceptability | ✓ | ✓ |  | ✓ | ✓ |  |  |  |  |
| Adoption | ✓ |  | ✓ |  |  |  |  |  |  |
| Feasibility | ✓ | ✓ | ✓ | ✓ | ✓ | ✓ | ✓ |  | ✓ |
| Fidelity | ✓ |  |  |  | ✓ |  | ✓ |  | ✓ |
| Costs | ✓ |  |  |  |  |  |  | ✓ |  |
| **Client Outcomes**  Satisfaction |  | ✓ |  | ✓ |  |  |  |  |  |
| Quality of Life (EQoL-5D)  Physical activity and sedentary behavior |  |  |  |  |  | ✓  ✓ |  |  |  |

^a^PROMS: Patient Reported Outcome Measures
