# Supplementary material for: Telerehabilitation’s Safety, Feasibility, and Exercise Uptake in Cancer Survivors: Process Evaluation
Source: JMIR Cancer. 2021 Dec 21;7(4):e33130. doi: 10.2196/33130 (PMC8768007; doi:10.2196/33130)
Supplement: Multimedia Appendix 2 [file cancer_v7i4e33130_app2.docx]

Supplementary file 1. Interview schedule

| **Proctor Domain** | **Evaluation Question** | **Interview question** |
| --- | --- | --- |
| **Acceptability and satisfaction** | **Is the intervention acceptable to clinicians?**  **Is the intervention acceptable and appropriate for delivery  in this setting?** | *Clinicians*  What was your experience of delivering/supporting this intervention?  Did you enjoy it?  How comfortable were you delivering/supporting it?  How did telerehabilitation “fit” with the ambulatory oncology rehabilitation model?  *Managers*  What was your experience of having this intervention delivered within your service?  What feedback did you receive from staff or patients related to the intervention?  How appropriate was it for therapists to provide telerehabilitation in this setting? |
| **Adoption** | **How likely would it be that clinicians/services would choose to implement this intervention beyond COVID-19?** | *Clinicians*  After the trial finishes, do you think you will keep using telerehabilitation for people with cancer?  Do you think you would apply this intervention future work in other settings?  Do you think managers would support ongoing implementation of telerehabilitation? Why or why not? *Managers* After the trial finishes, will you support the ongoing use of telerehabilitation with patients recovering from cancer?  How could this this intervention be applied in other settings in the future?  Would you be prepared to advocate for telerehabilitation within your health service? Why or why not? |
| **Feasibility and safety** | **Can the intervention be successfully delivered in this setting?** | *Clinicians*  What factors supported delivery of the intervention in this setting?  What were the positives and challenges?  Do you feel that you were able to deliver the intervention as was intended, and as instructed?  How safe was telerehabilitation?  *Managers*  Overall, how successful was the telerehabilitation model? (what worked well/not so well)  Were there barriers/challenges that needed to be overcome to put it into practice?  What barriers do you foresee if implementing telerehabilitation beyond a trial setting? |
| **Fidelity** | **Was the intervention delivered according to evidence?** | *Clinicians only*  How well were you able to deliver the intervention as instructed? |
| **Costs** | **What costs were involved in delivering the intervention?** | *Managers only*  What costs were involved in delivering telerehabilitation?  How well was the department able to support costs involved in delivering telerehabilitation? |
