# Supplementary material for: Telerehabilitation’s Safety, Feasibility, and Exercise Uptake in Cancer Survivors: Process Evaluation
Source: JMIR Cancer. 2021 Dec 21;7(4):e33130. doi: 10.2196/33130 (PMC8768007; doi:10.2196/33130)
Supplement: Multimedia Appendix 3 [file cancer_v7i4e33130_app3.docx]

|  | **Yes/No** | **Comments** |
| --- | --- | --- |
| **For each patient** |  |  |
| HEP |  |  |
| Group exercise class |  |  |
| Group education class |  | If so, which topics; |
| Access information portal |  |  |
| Receive written handouts |  | if so, which topics; |
| Nursing assessment |  |  |
|  |  |  |
| **Random file audit of coaching sessions** |  |  |
| *Was there evidence of any of the following?* |  |  |
| Evocation of change talk  e.g.   - Confidence/importance ruler - Use of reflections - Use of affirmations - Patient coming up with behaviour change strategy on their own - Use of DARN CATs questions |  | Describe: |
| **Use of BHI (based on taxonomy)**  Goal setting (behaviour)  Review of goal setting (behaviour)  Barrier identification/ Problem solving  Prompt self-monitoring of behaviour  Model/ Demonstrate the behaviour  Plan social support/ social change |  |  |
|  |  |  |
|  |  |  |
|  |  |  |
|  |  |  |
|  |  |  |
| Other |  | Describe: |

Supplementary file 3: Criteria for fidelity audit
