# Supplementary material for: Telerehabilitation’s Safety, Feasibility, and Exercise Uptake in Cancer Survivors: Process Evaluation
Source: JMIR Cancer. 2021 Dec 21;7(4):e33130. doi: 10.2196/33130 (PMC8768007; doi:10.2196/33130)
Supplement: Multimedia Appendix 4 [file cancer_v7i4e33130_app4.docx]

Supplementary file 4. Selected staff interview quotes mapped to Proctor Model

| **Proctor outcome** | **Sub-theme** | ***Quote*** |
| --- | --- | --- |
| Safety | Clinician training  Balancing needs       Video advantage | - *“For myself I felt like I wasn't trained, to deliver telehealth. You had to pick it up and learn it really quickly so what we were giving our patients, you know was safe and effective but then that was kind of the tip of the iceberg and then all the way below was all these other systems and processes that we had to kind of get our heads around.”* *(Participant 3)* - *“In terms of the exercise prescription from a safety perspective, I was very conservative. (Participant 4)… it was kind of challenging to balance the safety but trying to push people at the same time.” (Participant 1)* - *“I think in terms of fitting with the model like the key difference of safety and clinicians being able to monitor or assess their technique or how they're responding to the exercise.” (Participant 3)* - *“As technology develops and if we have access to technology where we can remotely monitor people's heart rate accurately, use wearables and we can certainly increase the safety of the program and if you have that one-on-one, face-to-face for the first appointment to really set the parameters then it can definitely be a safer model of care (Participant 1)…Agreed. One face-to-face initially wouldn’t solve all the problems but it would offer a an extra layer.” (Participant 2)* - *“ I thought it was okay because we're on video conference in this lady had a home gym set up…proper pin loaded weights machine and so trying to talk her through doing RM testing was okay…it was just really hard to try and correct her technique…the next week she developed lymphedema.” (Participant 1)* - *“it's such a nice option because then you can see people virtually and if they’re struggling…so if they do fall or if they do have risk clinical risks, we can see them on the video and we can guide them.” (Participant 8)* |
| Acceptability | Pride and satisfaction | - *“It was satisfying to be able to deliver something when lots of other services had completely stopped but as we all know a cancer does not stop and the side effects of treatment do not stop…it was comforting to know that we had something to offer and it was the best we could do in a really awful situation…satisfying was a really good word, I wouldn't say it's fun.” (Participant 4)* - *“It was comforting to know that we had something to offer and it was the best we could do in a really awful situation, so if we hadn't got telehealth off the ground, I think I would have felt quite helpless knowing we couldn't help these people. So that was satisfying.” (Participant 4)* - *“I really loved the challenge of being able to do telehealth because it showed others that this could be done.” (Participant 3)* - *“They love it! I mean even my (other program) clinicians that are not part of the program they all see it as a positive…think all of them are quite, proud of what they've achieved, from the oncology team, and even the other chronic rehab teams.” (Participant 8)* |
|  | A rollercoaster | - *“it would be like a rollercoaster more than exciting… some moments we just go, I don't know if this is working but it was it was fun and even exciting [but] the group thing was the thing that I missed and we weren’t sure whether we're catching people and supporting people as well as we could have.” (Participant 2)* - *“when patients got it, they embraced it …and then we found the demographic of our patients was changing, we're getting more people with advanced cancer…increasing functional needs and there were patients that were essentially dying but we didn't necessarily know that and there's been several moments in the program where I've been like I can't do anything for this patient.” (Participant 1)* |
|  | Connections and teamwork | - *“we used to see those patients come in and make those connections at reception and they'd always say hello and things like that where you don't get that anymore (Participant 7)…Instead of yeah getting to know the patients we've just getting to know the names (Participant 6)… I would know nobody who was in the group now. I wouldn't even know how many people in there. I just admit them and discharge them.” (Participant 2)* - *“It’s not quite the same when you're through a screen…I think we've really missed being in a gym and having that energy of having people around us…on the other hand some patients have really benefited from the one-on-one interaction. That maybe that they wouldn't have got, in a group environment.” (Participant 1)* - *“I think these patients were feeling particularly lonely and isolated…the lockdown was long…normally cancer patients have a huge health care team, they weren't really seeing any of those people. So to see us every week they felt that connection there was there was a lot of counselling happening.” (Participant 4)* - *“From a physio perspective I personally felt like because we were seeing each week and we're having half an hour conversations with them. I actually grew quite close to some of my patients probably more so than I would if I was in a group environment.” (Participant 1)* - *“I felt that slightly removed from the process outside...Like you were kinda not part of it anymore.” (Participant 2)* - *“The group thing was the thing that I missed (Participant 2).”* |
|  |  | - *“Click and call and then have to immediately pick up a new patient that's going to cry at you. It's intense…It’s really draining…(Participant 4)…I felt like a telephone operator some days, which, like is completely different from what I'm used to working as a physio.” (Participant 3)* - *‘We didn't really anticipate how intense that would be and so that we didn't we also were quite isolated from each other…we probably could have planned better to have some informal debriefing catch up (Participant 2)”* - *“We would have back to back to back patients and not see each other all day even at lunchtime...I'm the type person that does get energy from the people being in the room and those informal catch ups in the morning and at lunchtime [are valuable] so when you’re literally locked in a room all day…” (Participant 1)* |
| Adoption | Rapid implementation    Facilitators | - *“It was quite a rapid COVID force transitioning to this model and, incredible how quickly it got off the ground, that was, in terms of reviewing and looking at the program it was amazing how hurdles were jumped, and things were put in place really quickly.” (Participant 2)* - *“Very steep learning curve…These dormant models have been sitting there haven't been funded and pushed in and rolled out and all of a sudden there was an urgency to do it.” (Participant 3)* - *“Everyone in this team is quite computer orientated they weren’t scared...they pre-planned.” (Participant 7)* - *“There was very much a can-do mindset, from the team, I think across the board (Participant 2)… There was positives about it from everyone not the there wasn't a negative person.” (Participant 7)* - *“Was driven by people who were given time and permission to do it.” (Participant 2)* - *“This team thing comes up again, we're quite a tight closely knit team that are very good at communicating with one another which meant that communicating change was really easy and being able to provide education was useful.” (Participant 1)* - *“[referring to other staff] you felt like you might have been a little bit detached but even little things like sending the emails was critical to the success of the program so as a team we work really well.” (Participant 1)* - *“I think [physiotherapist] drove it because she had that passion for research…. And that’s how it took off.” (Participant 8)* - *“We should have strong team leaders that advocate for it and then they would sustain its own (Participant 8).”* |
|  | Expansion beyond oncology | - *“We did try and roll this model out across other they chronic rehab programs that wasn't quite as effective.” (Participant 1)* - *“There wasn't much uptake from the other programs…we were hoping that the staff would it would rub off on to them but it didn’t quiet work out. We are still looking at, expanding to those programs but that might take a while.” (Participant 8)* - *“Type of patients that they [other programs] have a lot older, sicker type patients and they’re not technologically advanced like some of the younger and fit patients and most of the patients in onc rehab.”(Participant 8)* |
|  | Future model | - *“It would be good to have some kind of hybrid model with the majority of centre base and I get more jobs satisfaction from center-based face-to-face.” (Participant 4)* - *“…I can see the benefit for people that live more rural or remote areas or that are severely immunocompromised …to give them an option is a gift…but my preference is the face to face.” (Participant 3)* - *“The way the program is currently resourced it's difficult to see how you can deliver a effective telerehab model and a face-to-face model…(Participant 1) I think definitely I would love to offer telehealth as an additional service but then I would like clinicians and staff to be well supported by the organization and management too… I don't think we can deliver telehealth effectively if we don't have the training or the support or the equipment to run it well.” (Participant 3)* - *“The benefits of the future is it's there's a model there that we know works is as it works and it can be, broadened, transitioned or expanded.” (Participant 2)* - *“In addition to what we already offer, we can do three ways. The third option will be telehealth which is a nice option…It is a sustainable model.” (Participant 8)* |
|  | Resource intensive | - *“That it is actually quite a resource intensive model of care… it's difficult to see how you can deliver an effective telerehab model and a face-to-face model… if you get 24 people coming through a day in a group like the maths just kind of makes sense that it is more efficient.” (Participant 1)* - *“We thought we would do doing well just to be seeing patients during COVID…There was this overarching feeling of pressure, of to increase the throughput so to speak, overtly or not.” (Participant 2)* - *“It was either do that [online group] or do one-on-one and I don't think we had enough resources to do the one-on-one. It's a limited program. So I think doing it in this way you capture more patients.” (Participant 8)* |
| Feasibility | Service provision and accessibility | - *“What we were giving our patients, was safe and effective but then that was kind of the tip of the iceberg and then all the way below was all these other systems and processes that we had to kind of get our heads around and work together as a team to implement.” (Participant 3)* - *“I always wondered how many people were actually looking at it and getting benefit [from emails] because in a group you can ask people how were you last week after you've done your exercises.” (Participant 6)* - *“It was one answer to what a lot of our patients have been saying in the past, they complain about going to appointments finding parking the time taken before and after appointments, it just takes out the whole day for them…so something like a telehealth appointment, you could see that they could just log in from home, they were a lot more relaxed.”(Participant 3)* - *“I think generally there's less DNAs because, they've got the convenience of their own home.” (Participant 1)* - *“We were seeing patients that who might have been on treatment and feeling atrocious, they would never have come to a group… whereas even if they didn't necessarily feel a hundred percent to do a telehealth consult and they haven't logged we could call them up and they'd still be happy to have a chat on the phone. So they were still getting something whereas maybe they weren’t before.” (Participant 1)* - *“It’s such a good model to have in these trying times. It’s just another option for patients to have their exercise.” (Participant 8)* - *“And the fact that they could combine different models as opposed to just offering the one option if it’s just that much more attractive for them.” (Participant 2)* |
|  | Infrastructure | - *“At the start I was probably more set up for it at home then at work when I came to work and delivered telehealth at but there were all these other kind of logistical issues that I had to deal with like finding a space to actually set up my telehealth or having the equipment at work to even have a good video call.” (Participant 3)* - *“I think it highlighted what resources we already had that we could use and, so it was great to know that we had something…It also kind of showed what we didn't have or what we lacked.” (Participant 3)* - *“I think our internet could probably be a lot better…this building…there is a list of rooms that have great internet…if your lucky to book that room well congratulations. If you miss it…”(Participant 7)* - *“the headphones weren't compatible to the laptops…some of the desktops got cameras, and headphones but the software on the computer's don't run the run the camera.” (Participant 6)* |
| Costs |  | - *“If they understood the funding requirement to get the throughput they want. They couldn't possibly support it.” (Participant 4)* - *“I feel we need further kind of funding and resources and support from the organization to deliver this. So that the team feels kind of supported and each one knows each person knows its roles and is comfortable in this role.” (Participant 3)* - *“Minimal setup costs, in fact it was just business as usual cost-wise…cost wasn't any different …in fact it would have, would have been a little bit cheaper because we would never had to physically bring patients into the center and do all the manual types of program that we would have had to deliver.” (Participant 8)* |
| Fidelity | Conflicted about effectiveness | - *“There’s sort of a ceiling, of what you can offer we telehealth in terms of intensity of exercise…I would be more conservative in my prescription.” (Participant 4)* - *“It's just a different approach like I think there's more of a physical activity approach and meeting the COSA guidelines and reducing sedentary behaviour, but if we're thinking about exercise guidelines, it's hard to get a gauge on it as much because there's not as close as supervision.” (Participant 1)* - *“It's hard knowing that they might not get, as much benefit…because ideally they would push a bit harder but just from a safety perspective. I didn't want them to.” (Participant 4)* - *“I think we can deliver telerehab to oncology patients really effectively and sometimes even with better outcomes like through patient uptake and patient convenience and engagement.” (Participant 3)* |
