# Supplementary material for: Telerehabilitation’s Safety, Feasibility, and Exercise Uptake in Cancer Survivors: Process Evaluation
Source: JMIR Cancer. 2021 Dec 21;7(4):e33130. doi: 10.2196/33130 (PMC8768007; doi:10.2196/33130)
Supplement: Multimedia Appendix 5 [file cancer_v7i4e33130_app5.docx]

Supplementary file 5. Telerehabilitation program costs

| **Human resource** | **Cost** | **Human resource** | **Cost** |
| --- | --- | --- | --- |
| **Telerehabilitation Model** | | **Face-to-Face Model** | |
| 0.6 EFT Physiotherapist Grade 2 1032 hours, 10 months | $48,823 | 0.6 EFT Physiotherapist Grade 2 1032 hours, 10 months | $48,823 |
| 0.5 EFT Physiotherapist Grade 3 860 hours, 10 months | $31,469 |  |  |
| Nurse Grade 5  632 hours, 8 months | $42,286 | Nurse Grade 5  1120 hours, 10 months | $65,273 |
| Allied Health Assistant  1 hours/week  43 hours | $1,257 | Allied Health Assistant 3hrs/week  120 hours | $3,509 |
| Administration assistant  4 hours/week  172 hours | $5,088 | Administration assistant  4 hours/week  172 hours | $5,088 |
|  |  |  |  |
| **Training** |  |  |  |
| Health coaching workshop | $1,500 |  |  |
|  |  |  |  |
| **Equipment costs** |  |  |  |
| Laptop computer x 2 | $4396 |  |  |
| Headsets with microphone x 2 | $450 |  |  |
| Theraband | $129 |  |  |
|  |  |  |  |
| **Software costs** |  |  |  |
| Videoconferencing license | $300 |  |  |
| Home exercise program license | $56 |  |  |
| **TOTAL (AUD)** | $135,754 |  | $113,593 |
| **Number of Patients** | 123 |  | 81 |
| **Cost per patient** | $1104 |  | $1402 |

^a^Phoneline, wifi and HealthDirect and iLearn platform costs not included in calculation as they are existing organisation-wide resources

^b^Staff costs do not include on-costs
